# *Echinococcus granulosus* Infection and Options for Control of Cystic Echinococcosis in Tibetan Communities of Western Sichuan Province, China

**DOI:** 10.1371/journal.pntd.0000426

**Published:** 2009-04-28

**Authors:** Yu Rong Yang, Donald P. McManus, Yan Huang, David D. Heath

**Affiliations:** 1 Molecular Parasitology Laboratory, Queensland Institute of Medical Research, Brisbane, Queensland, Australia; 2 Ningxia Medical University, Yinchuan, Ningxia Hui Autonomous Region, People's Republic of China; 3 School of the Population Health, University of Queensland, Brisbane, Queensland, Australia; 4 Institute of Parasitic Disease Control, Sichuan Centres for Disease Control, Chengdu City, Sichuan, People's Republic of China; 5 AgResearch New Zealand Limited, HopKirk Research Institute, Grasslands Research Centre, Palmerston North, New Zealand; Universidad Peruana Cayetano Heredia, Peru

## Abstract

**Background:**

Human cystic echinococcosis (CE) is highly endemic in the Tibetan regions of Sichuan where most families keep guard dogs and where there are considerable numbers of ownerless/stray dogs. Strong Buddhist beliefs do not allow for elimination of stray dogs, and many strays are actually fed and adopted by households or monasteries. On account of the high altitude (3900–5000 m), pasturage is the major agricultural activity in this area. The harsh mountainous climate often leads to many grazing animals dying on the pasture at the end of a hard winter. The skin and some meat are taken, and the rest of the animal is left for scavenging birds and animals. The poor sanitation and hygiene, the Buddhist doctrine of allowing old livestock to die naturally, plus the unrestricted disposal of animal viscera post-slaughter may be responsible for the high prevalence of human CE in this setting.

**Methods and Findings:**

As part of a large collaborative control program for CE in Ganzi County, situated in the west of Sichuan Province, surveillance for *Echinococcus* infection in domestic dogs using a coproantigen method and necropsy of unwanted dogs was carried out prior to (in 2000) and after (in 2005) dog anthelminthic treatment (5 mg/kg oral praziquantal at 6 month intervals) to determine the efficacy of the treatment for control. The prevalence of *E. granulosus* only in dogs by necropsy was 27% and 22%, and prevalence of both *Echinococcus* spp. by necropsy was 63% and 38%; prevalence of both *Echinococcus* spp. by coproantigen analysis was 50% and 17%. Necropsy of sheep/goats (age <1 to 12 years) (prevalence of *E. granulosus* in 1–6-year-old animals was 38% and in 10–12-year-old animals was 70%) and yaks (age 4 years) (prevalence of *E. granulosus* was 38%) was undertaken to determine the baseline transmission pressure. Protoscoleces were only found in very old sheep/goats and yaks. Necropsy of dogs in the Datangma district indicated that there was no apparent significant change in the overall prevalence of *E. granulosus* in unwanted dogs after 5 years of 6-month praziquantel treatment. However, this was likely due to the number of dogs available for necropsy being too small to reflect the real situation prevailing. There was a highly significant decrease in *Echinococcus* prevalence after the 5-year treatment program shown by coproantigen-ELISA. This indicated a decreasing but continuing risk for re-infection of domestic and stray dogs. Genotyping of *E. granulosus* samples obtained from necropsied sheep/goats and yaks and from locally infected humans at surgery was carried out to determine the strain of parasite responsible for human infection. DNA genotyping indicated that only the sheep strain (G1) of *E. granulosus* was present in the study area.

**Conclusions:**

Considerable re-infection rates of *E. granulosus* among dogs indicated a high infection pressure from infected livestock in this region, most likely from older animals dying on the pasture. A combination of livestock vaccination with the Eg95 vaccine, which is effective against the sheep strain of *E. granulosus*, and dog anthelmintic treatment, thus targeting two critical points of the parasite life-cycle, would avoid the conflicts of religion or local culture and could achieve the goal of hydatid control in the long term.

## Author Summary

Human cystic echinococcosis (CE) is highly endemic in Tibetan regions of Sichuan. As part of a control program for CE in Datangma district, Ganzi County, necropsy of strays and coproantigen-ELISA of all dogs was carried out prior to and post-drug treatment to determine the efficacy of the treatment for control. Examination of sheep/goats and yaks was undertaken to determine the baseline transmission pressure to dogs. The necropsy results indicated no apparent significant change in the overall prevalence of *E. granulosus* in unwanted dogs after 5 years of 6-month treatment. In contrast, there was a highly significant decrease in *Echinococcus* prevalence in domestic/stray dogs after the 5-year treatment program shown by coproantigen-ELISA. This indicated a decreasing but continuing risk for re-infection of dogs resulting from high infection pressure from the numerous infected domestic animals. DNA genotyping indicated the presence only of the sheep strain (G1) of *E. granulosus* in the study area. A combination of livestock vaccination with the highly effective Eg95 vaccine and dog drug treatment, targeting two critical points of the parasite life-cycle, would avoid the conflicts of religion or local culture and achieve the goal of hydatid control in the long term in the area.

## Introduction

Cystic echinococcosis (CE), caused by ingesting the eggs of the dog tapeworm *Echinococcus granulosus* is distributed worldwide in both humans and ungulates [1], and is a major public health problem in western China [2],[3]. *E. granulosus* has a two-host carnivore-prey life cycle, which commonly involves dogs and farm livestock. A common source of infection for dogs is offal from infected livestock. Two types of hydatid cysts can be observed in the various intermediate hosts: fertile cysts, in which brood capsules containing protoscoleces are both joined to the germinal layer and are free in the hydatid fluid filling the cyst cavity, and infertile cysts, which may not produce protoscoleces or are immature and are therefore unable to continue the life cycle of the parasite [4],[5].

Tibetan communities in north-western Sichuan Province are hyper-endemic for echinococcosis [6]. Poor sanitation and hygiene, the Buddist doctrine of allowing old livestock to die naturally, plus the unrestricted disposal of animal viscera post-slaughter may be responsible for the high prevalence of human CE. As part of a large collaborative control program for CE, surveillance for *Echinococcus* infection in domestic dogs using a coproantigen method and necropsy of unwanted dogs was carried out in Datangma district, Ganzi County, which is situated in the west of Sichuan province, near the border with the Tibet Autonomous Region, prior to (in 2000) and post- (in 2005) 6-monthly dog anthelminthic treatment to determine the efficacy of the treatment for control. Necropsy of sheep/goats (age <1 to 12 years) and yaks (age 4 years) was undertaken to determine the baseline transmission pressure. As well, genotyping of *E. granulosus* samples obtained from necropsied sheep/goats and yaks and from locally infected humans at surgery was carried out to determine the genotype of parasite responsible for human infections. Previous examination of yaks ranging in age from 4 to 12 years old in the field and at local slaughter houses in Ganzi County, revealed a high prevalence of *E. granulosus* cysts of the common sheep-dog strain (G1 genotype) but no cysts with protoscoleces [7]. This is in contrast to other published abattoir records such as in western Iran [8] where fertile cysts were found in 75% of sheep and goats, 45% in cattle and 15% in buffaloes. Notably, the prevalence of human CE has been shown by ultrasound to be as high as 6% in Ganzi villages that are dedicated to pastoralism [9], although as no other intermediate host animals were examined for the presence of hydatid cysts during the earlier survey, it was unclear which animals were responsible for infecting dogs [7]. Among *E. granulosus*, there is recognised genetic heterogeneity whereby different strains have been shown to have different host preferences, morphology and other biological characteristics, likely to impact on control [10]. Although the common domestic sheep-dog strain (G1 genotype) is responsible for most human infections, there is substantial evidence to indicate that other strains also infect humans [10]. Genotyping of *E. granulosus* samples obtained from necropsied sheep/goats and yaks, and from locally infected humans at surgery in Datangma district was carried out to determine the strain of parasite responsible for human infection.

## Materials and Methods

### Study area and population

**Figure 1:**
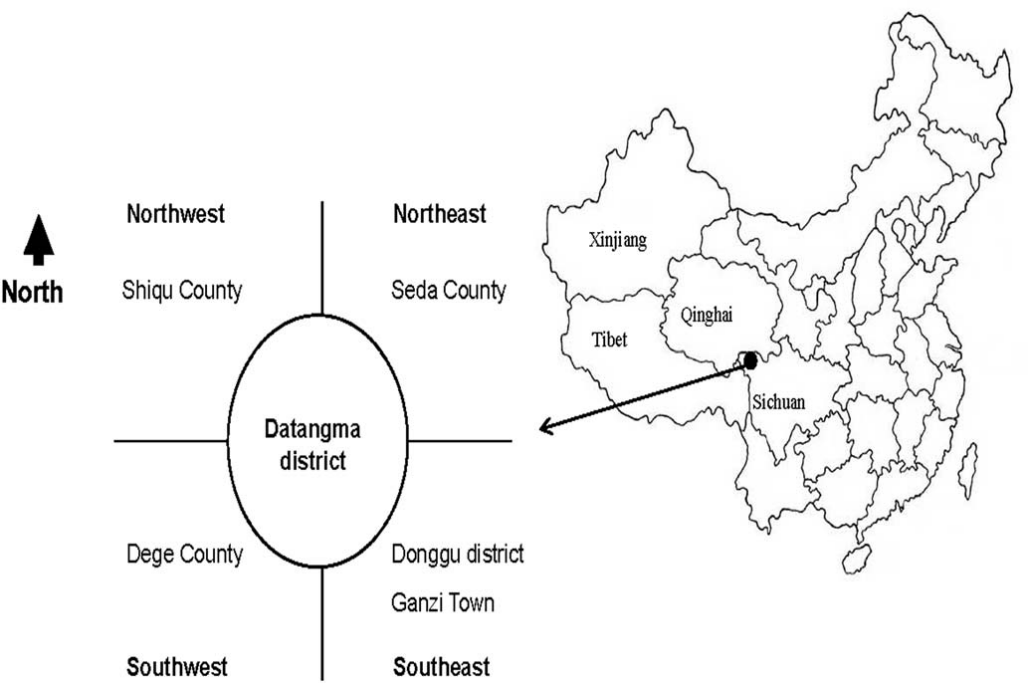
Map of China showing Datangma district in the northwest area of Ganzi Tibetan Autonomous Prefecture, Sichuan Province, China.

Datangma district, Ganzi County is in the north of Ganzi Tibetan Autonomous Prefecture, Sichuan Province, and consists of four townships (Cha-zha, Da-de, Ka-long and Cha-long) (Fig 1). Pasturage is the only agricultural activity in Datangma because of the high altitude (from 3900 m to 5000 m). Serious winter snow falls and summer flooding occur approximately every 5 years, and often lead to 50–70% loss of livestock in some villages/households. Within this study area, there are 89000 yaks, 25000 sheep, 8000 goats and 10000 horses (1999 records) which provide a primary income source for the local nomads. Most families keep at least one guard dog, and there are also large numbers of ownerless, stray dogs. The main source of water is the Daqu (moon) river that crosses the district from northwest to southeast; it provides sufficient water for human and livestock consumption, but there is no formal drinking water system. The water in the river is not clean as various items including dung, and yak, sheep and even human cadavers are thrown into it. Most of the households collect water several times a day for personal use and drink it without boiling. The population is composed primarily of transhumant herdsmen, who move their animals between winter pasturelands, now associated with fixed settlements, and higher altitude summer pasturelands not associated with fixed settlements. Hygiene and sanitation are extremely poor. In addition, people live in close proximity to both owned and unwanted domestic dogs.

### Dog treatment

All owned (4263) or stray dogs (1500–2000) in the study area were to be treated with praziquantel (PZQ) twice a year from the year 2000 onwards, at the end of spring and the end of autumn. It was not difficult to treat stray dogs because they were usually close to households or monastries and were always looking for food. Each dog received a 200 mg pill in tsampa (a mixture of barley meal and ghee), and was observed to make sure that the pill was ingested. At a recommended dose of 5 mg/kg, the 200 mg pill is sufficient for a 40 kg dog, and even the large Tibetan mastiffs would not exceed this weight.

### Dog coproantigen ELISA surveys

The dog coproantigen ELISA detection method used both prior to and following treatment of all dogs in the study area has been described [11]. On six occasions (Sept./2000, Sept./2003, May/2004, Oct./2004, Apr./2005, Oct./2005), faecal samples were collected from dogs from 20 selected households from each of 29 villages surveyed. They were allocated a number (1–580).

### Dog necropsy procedures

Unwanted dogs were euthanized humanely by feeding them a pill that contained para-amino propiophenone (PAPP). The small intestine was removed and opened longitudinally. After visual inspection of the surface of the intestine, the areas containing worms were placed in closed bottles of 0.85% (w/v) NaCl (saline) and vigorously shaken. Released worms were fixed in 4% (v/v) of 40% (v/v) formaldehyde in saline, either before or after relaxing overnight in tap water. *E. granulosus* worms were identified by morphology and were differentiated from *E.multilocularis* using light microscopy according to WHO Guidelines [12]. Other taeniid cestodes present in the necropsied dogs were identified based on established morphological criteria [13]–[15]. The only two *Taenia* species found were *Taenia serials* and *T. pisiformis*. It is possible that *T. multiceps* was present and could not be differentiated from *T. serialis*. However, there was no record in this region of *T. multiceps* metacestodes in sheep or goats, and also, surprisingly, no *T. hydatigena* or *T. ovis*.

### Sheep/goat/yak necropsy procedures

Yaks were observed after slaughter at Ganzi County abattoir. Sheep and goats were usually home-killed and were not available for necropsy inspection. The assistance of village and County cadres was sought in order to obtain young (4 years old) and older aged yaks (17–18 years old) or sheep and goats (aged 10–12 years). Animals were killed humanely by their owners, according to the Tibetan Buddhist custom. The head was presented, together with lungs and liver, so that an estimate could be made of the age of the animal. Yaks were aged from horn growth rings while sheep were aged by inspecting the number of incisor teeth and the degree of tooth wear. Organs were palpated and macroscopic cysts removed. The organs were then sliced at 2 mm (liver) or 4 mm (lungs) intervals to discover any small cysts. Cysts were dissected from liver or lung tissue. The cysts were then incised, and inner membranes and any brood capsules containing protoscoleces were rinsed in saline and then fixed in 95% (v/v) ethanol. Fixed samples were transported to the Australian laboratory for genotype analysis.

### Parasite sample collection and DNA analysis

*Echinococcus* cysts from sheep, goats and yaks were obtained from the study area as described above. An individual isolate represents parasite material collected from a single hydatid cyst. Human cystic material from patients of Datangma district was collected during surgery at Ganzi County Hospital. All cystic samples were examined by microscopy and isolates from sheep, goats, yak and humans that had protoscoleces and/or germinal membranes present were analysed by DNA sequencing of the *atp6* gene [16].

### Statistical analysis

Chi-square values and 95% confidence intervals were calculated using Epi-Info (Centres for Disease Control and Prevention, Atlanta, GA) for analyses of prevalence. Comparisons between groups were performed using chi-square of Fisher's exact tests. The level of statistical significance was set at P = 0.05, unless otherwise stated.

### Ethics statement

This study was reviewed and approved by Sichuan CDC, Sichuan Provincial Ethics Committee and the Institutional Review Board of the Ethics Committee of the First Teaching Hospital of Xinjiang Medical University as well as the Tibetan community representatives. Written informed consent was obtained from all participants before commencement. All animal work was carried out with the approval of AgResearch New Zealand limited, Wallaceville Animal Research Centre Animal Ethics Committee.

## Results

### Structure of the dog and livestock populations in Datangma district

#### Dogs

Most households kept 1–2 dogs; male dogs (70%) were preferred to bitches (30%). The main food of the dogs was the same as humans (barley plus ghee). Some nomads indicated they had seen the dogs eating hares, pika and rodents, mainly *Lepus oiostolus*, *Ochotona* curzoniae and/or *Microtus* spp. Dogs also ate the offal and remains of slaughtered or dead livestock, especially in the slaughtering season (November) and after a harsh winter (March). The nomads from this area treat their unwanted dogs and old or wounded dogs as free animals, and they never kill dogs because of their religious beliefs. Accordingly, there were a large number of unwanted dogs present. For the coproantigen survey one dog was sampled from each of 580 households (29 villages and 20 households per village). The same household was sampled each time, and in most cases it was the same dog. Some dogs died and were replaced. For the praziquantel treatment every dog in every household in every village, and all the unwanted dogs in the vicinity of each household received the pill in a small ball of tsampa (barley meal mixed with butter oil).

#### Livestock

In 2000, livestock in Datangma District comprised 89,000 yaks and 33,000 sheep/ goats. The yak herd structure comprised approximately 50% males, which were mostly castrated for transportation. Roughly similar numbers of males and females were in each age group ranging from 1–17 years. A few yaks were older than 17 years. Between the ages of 5 and 11 years, females might have one calf every 2 years. Most sheep/goat flocks had equal numbers of males and females with ages ranging from 1–12 years, with a few as old as 14 years. An average of 30% of females gave birth each year. The remaining females were too young or were past breeding age.

### *Echinococcus* prevalence

#### Prevalence in dogs

Dog necropsies were undertaken in 2000 and 2005. In 2000, necropsies of 22 unwanted dogs took place prior to the commencement of 6-monthly treatment of all dogs with praziquantel. The overall baseline prevalence of *E. granulosus* was 27% and that of *E. multilocularis* was 36% (Table 1). One dog had about 100,000 *E. multilocularis* worms, and others had 500–1000 *E. multilocularis*. All *E. granulosus* infections ranged from 10–50 worms. The prevalence of *Taenia* spp. (*T. serialis* and/or *T. pisiformis*) was 32%; 23% of dogs had no worms (Table 1). Two out of the 9 positive dogs had large numbers of *E.multilocularis* worms. The 5 dogs with *E.granulosus* worms had from 4–100 worms. The second series of necropsies took place at the end of 2005, 5 years after implementation of the control program; twenty-six stray dogs were necropsied, with an overall prevalence for *E. granulosus* of 22% and for *Taenia* spp. of 16% (Table 1). The prevalence of *E. granulosus* and the *Taenia* spp. decreased but the changes were not significant. Possibly, all of these were new infections, because all dogs in the region, wanted or stray, had been treated with praziquantel at the end of the previous autumn. There was a significant decrease (P<0.05) in *E. multilocularis* prevalence (16%) and a significantly increased percentage (54%) of dogs without worms (P<0.05).

**Table 1:** The prevalence of *Echinococcus granulosus* in dogs determined by necropsy and coproantigen surveys in 2000 prior to the commencement of a 6-monthly anthelmintic dosing program and 5 years later in 2005, Datangma district, Ganzi County.

Dog surveys	2000	2005	Significance
**Necropsy: intestinal cestodes present**	**(n = 22)**	**(n = 26)**	**(P-value)**
*E. granulosus only*	27%	16%	0.33
*E. granulosus* and *Taenia*. spp.^#^	0	4%	—
**Sub-total ** ***E. granulosus***	**27%**	**20%**	**0.42**
*Echinococcus multilocularis only*	18%	12%	0.33
*E. multilocularis* and *Taenia* spp.^#^	18%	4%	<0.01*
**Sub-total ** ***E. multilocularis***	**36%**	**16%**	**<0.05** *
**Total ** ***Echinococcus*** ** spp.**	**63%**	**36%**	**0.14**
*Taenia serialis* and/or *T. pisiformis only*	14%	8%	0.29
**Total of Taenia spp.** ^#^	**32%**	**16%**	**0.31**
Dogs with no worms	23%	54%	<0.05*
**Coproantigen detection** ^∧^	**(n = 580)**	**(n = 580)**	**(P-value)**
Coproantigen-ELISA positive for *Echinococcus* spp.	50%	17%	<0.01*

n, number of dogs.

*: Significant change between 2000 and 2005.

**∧:** Test does not differentiate between *E. granulosus* and *E. multilocularis*.

# *Taenia* spp. found were *T. serialis* and *T. pisiformis*.

In addition, the dog coproantigen positivity rate showed a constantly decreasing trend from 50% in 2000 prior to any treatment compared with post-treatment of 35% in September, 2003, 30% in both May and October, 2004, and 26% in April and 17% in October, 2005.

Comparing the coproantigen positivity rates, there was a highly significant difference in coproantigen positivity in dogs surveyed prior to treatment in 2000 compared with animals after 5-years of 6-monthly praziquantel treatment in 2005 (P<0.01) (Table 1).

#### Prevalence in intermediate hosts

In 2003, 15 goat and 19 sheep livers and lungs from animals up to the age of 6 years were examined; 3/15 goats and 10/19 sheep were infected (Table 2). The maximum diameter of cysts was 15 mm, but most had diameters of 2 to 3 mm. None of the cysts contained protoscoleces. *E. granulosus* prevalence appeared to increase with age. In 2004, seventeen sheep/goats aged 10–12 years were necropsied (Table 2). Within this older-age group, there were 6 animals with infertile cysts and 6 animals with fertile cysts having protoscoleces. There was an average of 5.8 cysts per infected animal.

**Table 2:** Hydatid prevalence in sheep/goats by age in Datangma district, Ganzi County.

Age group	Number infected/Number uninfected	Prevalence
<1–6 years^a^	13/21	38%
10–12 years^b^	12*/5	70%
Difference between the two age groups (P value)		0.03
RR (95% Cls)		0.54 (0.32–0.92)

a Examined in 2003.

b Examined in 2004.

*: Six animals had infertile cysts and 6 others had fertile cysts; there was an average of 5.8 cysts per infected animal.

Regarding larval *E. granulosus* infection in yaks, 100 four-year-old yaks were surveyed in the field and 38% had cysts. Of the infected yaks, there was an average of 8 cysts per infected animal (Table 3). Under macroscopic observation, both “apparently multilocular” and unilocular cysts were often found in the same host. Both these cyst types had been genotyped previously by *atp6* gene sequence analysis as G1 [7]. The “apparently multilocular” cysts were actually overgrown germinal membrane filling a space enclosed by a thick cyst wall. Different cyst sizes were also often observed in the same host. Among 305 cysts, the majority (79%, 241/305) were in the size range from 2 to 20 mm in diameter (Table 3). The “potentially-fertile” cysts were only found with cyst diameters larger than 21 mm, and the frequency of the “potentially-fertile” cysts increased with cyst sizes (Table 3). A “potentially-fertile” cyst is defined as one containing clear fluid, an intact germinal membrane that was not convoluted, and a thin host-derived adventitial membrane. Yak 2 (17 years old) (Table 4) had 2 lung cysts of 14 and 8 cm diameter. Both cysts contained large numbers of protoscoleces.

**Table 3:** The characteristics of hydatid cysts in yak by cyst type and size among 100 four-year-old necropsied animals, Datangma District, Ganzi County.

Cyst type*	Prevalence
Multilocular cysts^a^	55.5%
Unilocular cysts^a^	60%
**Cyst diameter (mm)**	**Numbers (%) of “potentially-fertile”/“unfertile cysts”**
2–20	0/241 (0%)
21–50	7/47 (13%)
≥51–≤90	8/2 (80%)
Total	15/290 (4.9%)

*: The survey involved 100 four-year-old animals. There were a total of 305 cysts among the infected yaks (38% prevalence). Both multilocular and unilocular cysts were often found in the same host. Different sized cysts were also found in the same host. All the cysts that were genotyped by *atp6* gene sequence represented the *E. granulosus* G1 genotype, as reported [7].

a Mean of 8 cysts per infected animal.

**Table 4:** Substitutions in the *atp6* gene of the *E. granulosus* common sheep/dog strain (G1 genotype) isolates investigated in this study.

Host origin	Geographic origin	Substitutions in *Atp6 gene* (position)	Isolate description
Yak 2	Datangma	G/A (360)	Protoscoleces/germinal membrane from a 14 cm-cyst in lung
		G/A (360)	Germinal membrane from a 8 cm-cyst in lung
Yak 3	Datangma	No DNA available	A 2 cm-cyst from lung
Yak 23	Datangma	T/C (243), T/C (265)	Lung cyst
Sheep	Datangma	T/A (86)	One infected/25 necropsied sheep
Human 1	Ganzi Hospital	C/T (75)	Germinal membrane of cyst from liver
Human 2	Ganzi Hospital	T/C (45), T/A (50)	Germinal membrane of cyst from liver
Human 3	Ganzi Hospital	T/C (265)	Germinal membrane of cyst from liver
Human 4	Ganzi Hospital	No mutation	Germinal membrane of cyst from liver
Yak-liu 1	Not Datangma	G/A (360)	Lung cyst with protoscoleces
Yak-liu 2	Not Datangma	G/A (360)	Lung cyst with germinal membrane, no protoscolex from yak lung
Sheep-liu1	Not Datangma	G/T (23), T/G (24), C/T (26)	Cyst with protoscoleces from liver
Sheep-liu 2	Not Datangma	T/A (43), T/C (73), G/A (360)	Cyst with protoscoleces from liver
Sheep-liu 3	Not Datangma	G/A (360)	Cyst with germinal membrane from lung

Not Datangma; isolates collected from other counties in Sichuan province close to Datangma district, Ganzi County.

### Sequence analysis of the *atp6* gene

Except for one yak isolate (yak 3; small (2 cm) cyst diameter, with no germinal membrane or protoscoleces present), all samples collected from animal intermediate hosts (sheep, goats and yaks) and humans were amenable to DNA typing by mitochondrial *atp6* gene sequencing. If protoscoleces were not present, the typing was done on DNA extracted from germinal membrane. All samples were typed as the G1 genotype of *E. granulosus*; some minor mutations in the sequences obtained were apparent as shown in Table 4. These substitutions were distributed across the length of the 513 bp fragment examined. The sequence analysis allowed the definition of 8 different types of *atp6* sequence represented by variation at 13 different nucleotide positions listed in Table 4. Of these, there were 6 isolates with variation at position 360 of the *atp6* gene. Seven isolates had substitutions at positions 23, 24, 26, 43, 45, 50, 73, 75, 86, 265 and 243. Isolates with a silent substitution at position 360 originated only from animal hosts; none of the human isolates had the mutation. All the *E. granulosus atp6* sequences obtained were translated into open reading frames thus eliminating the presence of pseudogenes.

## Discussion

The most reliable method for diagnosis of *Echinococcus spp.* in definitive hosts is by necropsy, because worm burdens can be accurately estimated and parasites collected for identification [17]. However, necropsy usually results in a biased sample, in that only unwanted dogs can be necropsied. Coproantigen detection of *Echinococcus spp.* in canine hosts has shown great promise [11] and provides a complementary method for diagnostic and surveillance purposes. Necropsy results prior to the start of the control program undertaken in the Datangma district study area in 2000 showed that only 23% of dogs had no worms, with prevalences for *E. multilocularis* and *E. granulosus* being 36% and 27%, respectively. Two *Taenia* species (*T. pisiformis* and *T.serialis*) with a similar transmission life-cycle to *E. multilocularis* (hares necropsied around townships often had *E. multilocularis* cysts in their liver), were found in 32% of dogs, and these were probably acquired by animals eating infected hares or rodents.

Praziquantel is currently the most effective anthelmintic available for echinococcosis control in carnivores [18],[19]. Mathematical models have been developed to describe the transmission dynamics and more recently to simulate control options [20],[21]. The lengthening of the treatment intervals to beyond the pre-patent period for *Echinococcus* spp. can be effective because the mean time to re-infection is often considerably longer than six weeks [20],[21]. Necropsy of dogs in the Datangma district indicated there was no apparent significant change in the overall prevalence of *E. granulosus* in unwanted dogs after 5 years of 6-monthly praziquantel treatment. The intensities of infection also did not appear to differ between the two sampling periods. However, this was likely due to the number of dogs available for necropsy being too small to reflect the real situation prevailing. The highly significant difference in *Echinococcus* prevalence after the 5-year treatment program shown by the coproantigen-ELISA indicated a decreasing but continuing risk for re-infection of domestic and stray dogs, and therefore of livestock.

In intermediate hosts, cysts of *E. granulosus* are usually detected at post-mortem abattoir examination of the viscera. Although this can provide important epidemiological data, which can be used to define likely echinococcal infection pressure [22]–[24], the main disadvantage of the approach is that samples obtained at slaughterhouses are potentially biased because this material is not generally accessible to dogs. In Tibetan areas, some animals are slaughtered (usually late autumn) for meat, but most old animals are allowed to die naturally. It is very difficult to necropsy these animals so that information about the *E. granulosus* infection status of very old animals is hard to obtain due to the strong Buddhist religion and life-style.

Interruption of the echinococcal parasite lifecycle in intermediate hosts has been shown to be important [25]. A vaccine for animal intermediate hosts with the Eg95 vaccine has been shown to be highly effective as an intervention against the dog/sheep (G1 genotype) strain [26]–[29]. The hydatid isolates examined here and previously from Ganzi indicated that only the G1 strain of *E. granulosus* is present and is responsible for human CE prevalence in this and other hyperendemic regions of North West China [3],[6],[7],[9],[16],[30]. Some minor genetic polymorphism in the *atp6* sequence was evident in a number of the isolates, with the most frequent change being a substitution (G/A) at position 360 compared with the G1 reference sequence in isolates collected from the majority of hosts except humans. Whether these polymorphisms in the *atp6* sequence reflect any biological or public health relevance for control may require further investigation on a larger scale, since genetic variation may affect infectivity and pathogenicity of *E. granulosus* [10],[31],[32].

The genotyping results are highly relevant in terms of control as livestock vaccination with the Eg95 vaccine would not only be beneficial in increasing the value of livestock production, but would also prevent the transmission of the parasite to dogs. The vaccine only prevents new infections and does not eliminate cysts already present. Thus, it would take a number of years before all the previously infected livestock were removed from the population. A modelling study has suggested a high probability of success even if anthelmintic treatment is only given every 6 months to 60% dogs with as few as 60% of sheep vaccinated [25]. This illustrates the cumulative effect of controlling the parasite at more than one point in its life cycle and may indicate the most promising means of control for this region. A control strategy consisting of a combination of livestock vaccination by using Eg95 and routine anthelmintic dog treatment would be particularly useful in this low income rural area where CE is so highly endemic, where resources are scarce and continual reintroduction of *E. granulosus* through sylvatic cycles or from neighbouring counties, provinces or even countries are constant threats. Unfortunately the study environment also has high transmission of *E. multilocularis*, which accounts for approximately 50% of human infections. The Eg95 vaccine would not affect the transmission of *E. multilocularis*. This will make overall control of echinococcosis more difficult. Control of *E. multilocularis* will require greater attention to hygiene and to more frequent treatment of dogs with praziquantel. Also, until the coproantigen test can be made specific, surveillance using this test will include both species of *Echinococcus*. Nevertheless, there did appear to be some encouraging progress made during the 5 years of 6-monthly dog treatments in Datangma district.

Many studies have illustrated that the development rate of *E. granulosus* cysts corresponds with the longevity of the intermediate host and that large cysts, 10–20 cm in diameter, are generally fertile [33]. In our observations, the frequencies of “potential-fertile” cysts increased in lesions with a diameter>21 mm in 100 four-year old yak necropsied. In the Tibetan region *E. granulosus* also appears to mature towards the end of the natural lifetime of its intermediate hosts. The fertile cysts were only found in sheep/goats around the age of 10–12 years (Table 2) and in very old yaks, possibly because the drop in immunity associated with aging allows the cysts to mature and develop protoscoleces. This finding is significant for future hydatid control in Tibetan areas and it might seem logical to begin a control program by removing all old or unproductive animals. Such a recommendation, however, though good for the economics of pastoralism, may change the farming practices that Tibetans have established over a thousand years of grazing animals on the high plateau. The combination of livestock vaccination with dog anthelmintic treatment might be a more acceptable strategy than removal of stray dogs and old livestock. In this way, a control program would avoid the conflicts of religion or local culture, but could still achieve the goal of hydatid control in the long term. However, now that new epidemiological information has become available there are already suggestions that a program of hydatid control should include removal of old and unproductive animals [34] and ownerless stray dogs.
